# Daylight Photodynamic Therapy: At-Home Delivery

**DOI:** 10.3390/jcm13247745

**Published:** 2024-12-18

**Authors:** David Bajek, Andrea Lesar, Carol Goodman, Daniella Levins, Paul O’Mahoney, Marese O’Reilly, Susan Yule, Ewan Eadie, Sally Ibbotson

**Affiliations:** 1 School of Medicine, University of Dundee, Dundee DD1 4HN, UK; 2 Photobiology Unit, NHS Tayside, Ninewells Hospital, Dundee DD9 1SY, UK; 3 Optical Radiation Effects, UK Health Security Agency, Chilton OX11 0RQ, UK

**Keywords:** PDT, photodynamic therapy, daylight PDT, dPDT@home

## Abstract

This pilot study evaluated the design, usability, and practicality of the dPDT@home kit for treating actinic keratoses (AKs) on the face and scalp. The kit allowed patients to manage their treatment at home, reducing hospital visits and utilizing natural sunlight. While patients were very willing to use the kit again, further studies are required to evaluate outcomes and ascertain the need for additional improvements and support. **Background/Objectives**: Daylight photodynamic therapy (dPDT) is an established effective therapy for superficial mild-to-moderate actinic keratoses (AKs) on the face and scalp. In this project, we redesigned the delivery of dPDT using design principles and the concept of Realistic Medicine to create the dPDT@home kit. This user-friendly and environmentally conscious kit allows patients to manage their AKs at home, reducing the need for hospital visits and ensuring timely treatment to coincide with appropriate weather conditions and to prevent disease progression due to delays in diagnosis and treatment. The initial pilot phase of the study was to evaluate the usability and convenience of the practicalities of the dPDT@home kit. **Methods**: Patients were instructed to conduct two dPDT@home kit treatments approximately three weeks apart on suitable weather days. After a follow-up telephone consultation from the specialist PDT nurse following the first treatment, patients then completed an initial questionnaire (Questionnaire 1, Q1) to share their experience. A second questionnaire (Q2) was completed 3–6 months after their final treatment to assess treatment outcomes. **Results**: A total of 16 patients with AK on the face and/or scalp used the dPDT@home kit. Five patients formed an initial pilot group in 2020/21, whose feedback and involvement informed the final product for the larger group of eleven patients (2021/22). All patients reported no issues with receiving the kit or the pro-drug used in the treatment (Q1). Q2 had an 81.25% return rate, with an average willingness score of 8.9/10 to use dPDT@home again. However, patients expressed doubts about their confidence in the treatment’s efficacy, giving an average score of 6.9/10, with preferences leaning towards other treatments, such as hospital-based PDT or cryotherapy. **Conclusions**: The pilot deployment of the dPDT@home kit identified suitable patients and highlighted the need for comprehensive training and support for both patients and clinicians to deliver dPDT through this novel approach. The kit can reduce the number of hospital visits, but patients still require supervision, which can be provided remotely. The questionnaire outcomes emphasize the importance of setting patient expectations and taking a holistic approach to managing chronic field-change AK. Additionally, the kit’s recyclable components and reliance on natural sunlight promote sustainability and reduce patient travel. Further evaluation is required to determine cost-efficacy, safety, and the potential place of the dPDT@home kit in the therapeutic management of patients with this common and challenging condition.

## 1. Introduction

Photodynamic therapy (PDT) is a therapeutic approach that involves the use of a photosensitizing agent activated by specific wavelengths of light in the presence of oxygen to generate reactive oxygen species, leading to localized cell death. PDT has applications in oncology, dermatology, and infectious diseases due to its selective targeting and minimal systemic toxicity. The process requires delivery and accumulation of a photosensitizer in target tissues. Upon exposure to light, the photosensitizer absorbs photons and undergoes a photochemical reaction, producing cytotoxic reactive oxygen species that induce cell death and modulate the local immune response. PDT has demonstrated efficacy in treating superficial non-melanoma skin cancers, actinic keratoses, and various infections. Key studies include pioneering work by Dougherty and colleagues on PDT for cancer therapy [1], as well as research highlighting its mechanisms and clinical applications [2,3].

Daylight photodynamic therapy [4,5] (dPDT) is a form of PDT that uses natural daylight to activate a photosensitizer, which accumulates in skin after application of a topical pro-drug, primarily for the treatment of actinic keratosis [6]. This method is as effective as conventional PDT but is less painful and more convenient than conventional PDT, as it uses ambient daylight rather than an artificial light source [7].

For a guide of efficacy, we have previously shown [8] that, for dPDT, 55% of patients were reported as having a very good or a good response, whilst 45% had a moderate response (in this study, both very good and good refer to disease clearance over 75%, whilst moderate refers to clearance between 50% and 75%). In another recent study [9], our patients had responses of 30–32% excellent, 37–41% good, 18–26% moderate, and 7–9% no response (in this study, excellent refers to minimal remaining disease, good refers to more than 75% disease clearance, moderate refers to clearance between 50% and 75%, and no response refers less than 50% of disease clearance).

While some patients already self-administer dPDT, the treatment design has yet to be optimized. As this marks a significant shift in how the therapy is delivered, it is crucial to explore the most effective ways to integrate this approach into clinical practice.

In 2015, the Chief Medical Officer (CMO) for Scotland published a national report entitled “Realistic Medicine” (Calderwood C. Chief Medical Officer’s Annual Report 2014–15 gov.scot: Scottish Government; 2016) which challenged medical practitioners to improve patient care through the development of a more personalized approach, one in which patients are placed at the center of their care. Although service improvement has always been a key priority within the photodynamic therapy center, we increasingly wondered if we had an accurate understanding of our patients’ needs. In 2019, the PDT center hosted a patient workshop with patients who had previously received PDT treatment. The double diamond design concept was employed to inform the program which aimed to gain a better understanding of the needs of patients using PDT, underpinning the objectives of the concept of realistic medicine. Through collaboration with patients and staff, we identified three main treatment characteristics which the patients [10] weighted in equal importance in their PDT treatment. The established characteristics were (1) the importance of patient convenience, (2) treatment efficacy, and (3) lack of pain associated with treatment. These insights set the stage for the development of the dPDT@home kit.

We present our solution: the dPDT@home kit. This kit, designed to be user-friendly and environmentally sustainable, provides the potential to empower patients to self-manage mild-to-moderate AK at home. By enabling treatment outside hospital settings, an aspect which patients were shown to be keen on exploring [11], it minimizes the necessity for hospital visits, ensuring timely intervention to prevent AK lesions from progressing due to delays in diagnosis and monitoring. Our study focuses on the face and scalp, as these areas of the body are the licensed indication for daylight PDT (Metvix^®^ (Galderma, Evergreen House North, Grafton Place, London, NW1 2DX, UK) 160 mg/g cream), and our experience is that efficacy is not as good on other body sites.

## 2. Materials and Methods

In this section, we shall outline the development of the dPDT@home kit, followed by the process of gathering information from the patients who used it. This idea emerged during the COVID-19 pandemic to minimize, where possible, contact between patients with AK and multiple co-morbidities, with healthcare premises. As such, the project was registered with NHS Tayside Clinical Governance as quality improvement work and service evaluation.

### 2.1. Developing the PDT@home Kit (2020)

**Figure 1:**
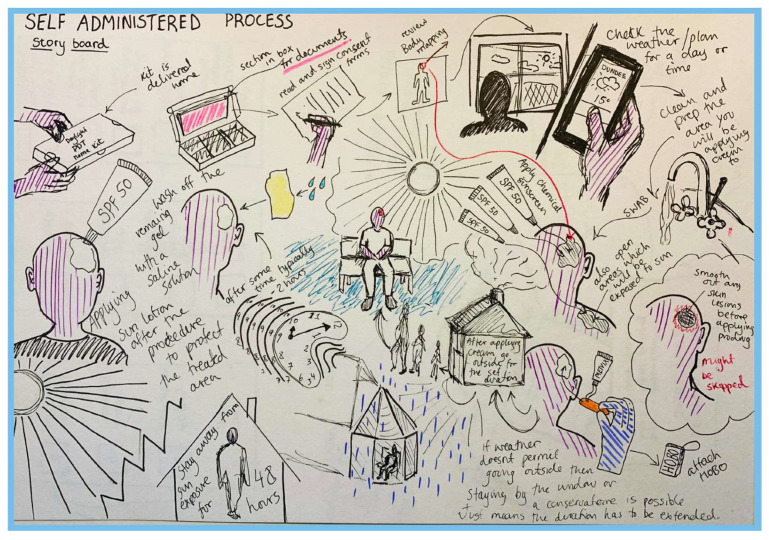
Patient-driven storyboarding conceptualizing the PDT @home kit.

The PDT@home kit is a robust carboard box, which contains all materials and information required for a patient to carry out dPDT at home, without having to come to the hospital. We followed a patient-led design philosophy and included patient feedback and focus-group sessions with prototype box designs. The complete patient journey was storyboarded, ensuring no elements of the treatment were missed in the design of the kit (Figure 1). The box was then produced and introduced to the clinic.

**Figure 2:**
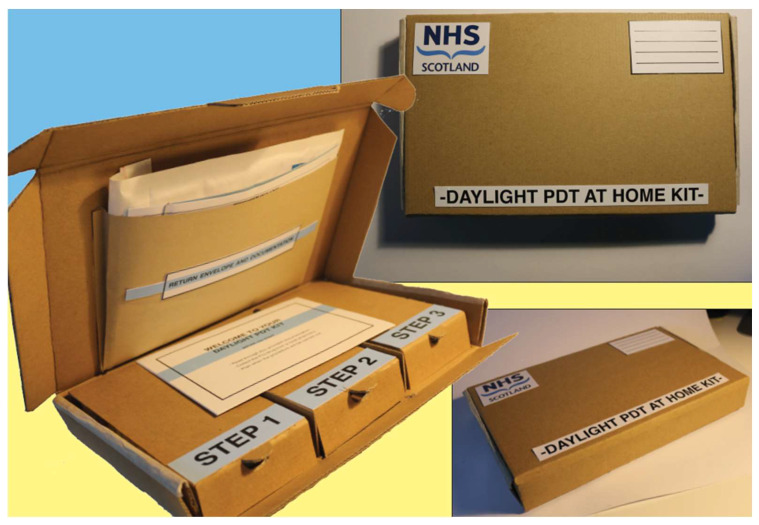
The dPDT@home kit, interior and exterior.

Specifically designed to be posted to and from the patient, the box has dimensions of 23 × 18 × 4 cm (length × width × height). Internally, the box is split into compartments, which facilitate the main steps of use (Figure 2).

**Figure 3:**
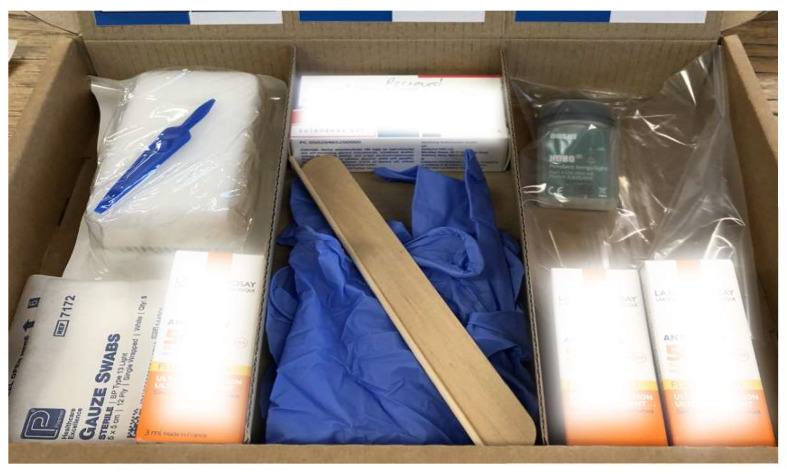
The contents of the dPDT@home kit.

The box is filled with all items required to carry out dPDT@home, split between the steps in which they are used. These include protective gloves, SPF50 Sunscreen, a HOBO pendant photodiode data logger (which logs their exposure to light over time), cleansing swabs, applicators, and a prescription for the pro-drug Metvix^®^ (Figure 3).

**Figure 4:**
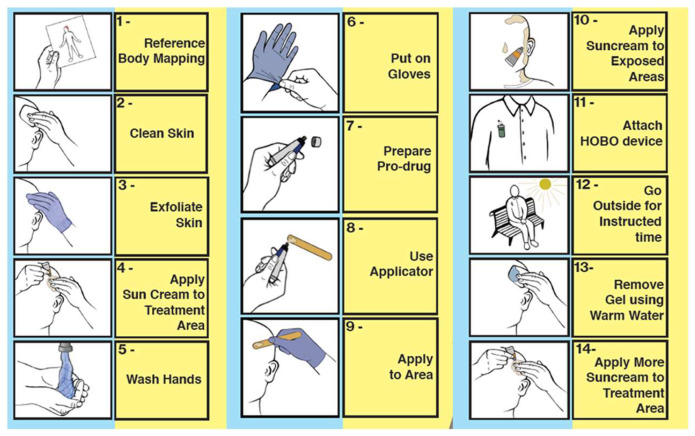
Step-by-step instructions for the dPDT@home kit.

Additionally, there are step-by-step instructions in the included card (Figure 4) that remind the patients of the order in which to carry out their treatment, and attached to the inside of the lid is an envelope for patients to return the HOBO data logger and treatment documentation.

Importantly, almost all components of the kit are recyclable, and patients were encouraged to recycle as much as they were able to in order to fulfil our desire for a product which meets our sustainability initiatives.

### 2.2. Participant Use of the PDT@home Kit

Patients referred by clinicians, mainly dermatologists and plastic surgeons, for dPDT to treat AKs attended the Photobiology Unit at Ninewells Hospital in Dundee for an initial clinical assessment. This assessment focused on AKs of the face and scalp, adhering to licensed indications for dPDT and specified inclusion criteria, followed by a discussion about the treatment. Regarding inclusion criteria, patients were selected based on having been referred for PDT and requiring treatment for active AKs. Such patients were to be considered able and were willing to undertake treatment themselves at home, accepting the need for them (or their carer) to follow instructions and be involved in their own treatment process. Treating clinicians independently made deviations from protocol based on the patient’s suitability for treatment. A joint decision between the patient and the clinical assessor determined whether the patient would undertake dPDT at home.

There were a small number of patients who we felt would struggle with self-administration of treatment; e.g., some patients would have struggled to apply the pro-drug on the difficult-to-reach sites, such as the scalp, due to mobility issues or difficulty viewing this site in a mirror—in these cases, a relative was trained. If a patient did not have someone at home to help with treatment, then we carried out conventional daylight PDT. We did have patients who declined the offer of dPDT@home, preferring to attend for conventional daylight PDT. Additionally, some patients were not suitable for self-preparation of the identified areas (using the supplied scrub brush), as their AKs were too severe—these patients attended for conventional PDT. All patients undergoing dPDT@home now attend the department for curettage of AKs, so we can better prepare the skin prior to them applying Metvix^®^.

After the initial consultation, participant information sheets were provided, and a training visit was arranged between the patient and the specialist PDT nurse at the Photobiology Unit. During this training visit, all aspects of treatment delivery and requirements were discussed, and the patient provided written informed consent for the treatment, as is our usual practice for routine PDT. Patients were trained in how to prepare their skin, apply sunscreen and the pro-drug, and undertake daylight exposure.

At the first visit, the treatment procedure is explained, including application of Metvix^®^ and sunscreen. We demonstrate the application of Metvix^®^ using, e.g., an emollient on the back of the hand so that the patient can visualize how much cream they need to apply. We also explain the possible side effects, aftercare, completion of the treatment recording sheet, and use/fitment of the HOBO device. Patients are given an information leaflet—contact details are reinforced should they need to contact us. If deemed necessary, the nurse performed surface preparation to loosen and remove scaling or crusting. The patient was then provided with two dPDT@home kits and a prescription for the pro-drug. They were instructed to carry out two treatments approximately three weeks apart on convenient days, with appropriate weather conditions, as advised by the specialist nurse. A review appointment was scheduled for approximately six weeks after the first visit, and a follow-up telephone consultation from the specialist PDT nurse was arranged after the first treatment.

At the review appointment, the treatment response was assessed, and if appropriate, a plan for further treatment was initiated. This involved providing another two dPDT@home kits, a prescription for the pro-drug, and instructions to carry out two more treatments approximately three weeks apart. A follow-up telephone consultation was arranged after the third treatment. Patients then attended a clinical review approximately 3–6 months after their final treatment.

Each data-logging light meter was calibrated against a photopic illuminance meter (VL-3701, Gigahertz Optik GmbH, Türkenfeld, Germany). The calibration light source was daylight, and the calibration was carried out on multiple occasions throughout the year. The model (described by O’Mahoney et al. [12]) was then used to convert the illuminance measurement into a PDT relevant protoporphyrin-IX (PpIX) weighted dose. This is the same technique used in our hospital-based daylight PDT service [8].

To ensure the patients are following the protocols with regard to the data-logger, the patients were asked to fill out a form (which is also used in hospital-based dPDT), writing down when they started and when they ended treatment. When we then read the data from the logger, we compared to determine if that timeframe corresponded to light-exposure data.

## 3. Results

This quality improvement project ran during the dPDT treatment season through spring and summer in NHS Tayside, from May 2020 to the end of September 2022. The design phase with patient input took place in 2020, a pilot group was involved in 2021, with the final evaluation group in 2022. During this period, 16 patients with diffuse or multiple AKs on the face and/or scalp undertook dPDT at home, ranging in age from 53 to 84 years. All patients treated the head and neck.

### 3.1. Pilot Group (Six Patients, 2021)

A pilot group of six patients (five male; one female) with median age 69 (range 53–74) years participated in 2021. The total number of treatments was 18, with a median of 3 treatments per patient (range 2–4), with a median protoporphyrin IX (PpIX) dose of 16 Jcm^−2^ (range 11–20 Jcm^−2^) and treatment time of 2 h 40 min (range 2 h 15 min–3 h). Four of the six patients had a good (>75% clearance) clinical response, with one moderate (50–75% clearance) and one poor (<50% clearance) response. The median pain score on a visual analogue scale of 0–10 was 1.5 (within range of 0–7.7).

Five of the six patients responded to a questionnaire on their experience of the dPDT@home kit, with questions ranging from the convenience and efficacy of the treatment to the recyclability of the kit, in line with our sustainability initiative. Four of the five responses said they had a good treatment result from the dPDT@home but “would have liked a bit better clearance”. One participant felt there was no change in the treatment area. These results correlate with the clinical assessment of the treatment area. All would be happy to have dPDT@home again, found the dPDT@home kit easy to use, and were confident performing their treatment at home. Four of the five respondents recycled all or some of the kit, but recycling the kit was very important to only two of the five respondents.

### 3.2. Expanded Group (11 Patients, 2022)

Following the successful pilot in 2021, the dPDT@home project was expanded for patient use in 2022. Eleven patients participated (nine male; two female), with a median age of 72 (range 54–84) years. This group included five patients from the 2021 group receiving repeat treatment, which reflects the chronic nature of AK and the requirement for continuous disease management. In total, there were 41 treatments in 2022. Three patients had three treatments, with the rest receiving four treatments. There was a median PpIX dose of 17 Jcm^−2^ (range 4–30 Jcm^−2^), which is similar to the median dose in 2021. However, 13 treatments (32%) had no dose recorded, compared with just 2 (11%) in 2021. The pain score was similar to 2021, with a median value of 1.3 (range 0–7.0). Nine of the eleven participants had follow-up appointments 3–6 months after the last treatment, with five recorded as good clearance (>75% clearance), one as moderate (50–75% clearance), and three as poor (<50% clearance). The patients with good clearance all had mild-to-moderate disease, whereas the poor responses were all in patients with moderate-to-severe AK. We define mild AK as “thin, just palpable”; moderate as “easily palpable”; and severe as “thick, crusted, hyperkeratotic”.

dPDT@home was rated highly overall (average 8.7 ± 0.90 on a scale from 1 (poor) to 10 (excellent), was rated highly convenient (average 8.8 ± 0.98 on a scale from 1 (not convenient) to 10 (very convenient), and rated was highly on keenness to repeat dPDT@home (average 7.8 ± 0.85 on a scale from 0 (not at all) to 10 (extremely keen)). Despite this, there was not a high level of satisfaction among the patients. Only two patients said they were fairly satisfied with the treatment, with five saying they were neither satisfied nor dissatisfied, and one being fairly dissatisfied. There were no responses from the remaining three patients. As the questionnaires were anonymized, we are unable to determine if the satisfaction corresponded with the clinical outcome recorded. Eight patients responded that they might (five) or would (three) prefer other treatments for their sun-damaged skin. Lower satisfaction ratings could suggest that, at home, daylight PDT may not be the optimal treatment for those with moderate-to-severe AK, and alternatives such as 5-Fluorouracil or conventional red-light PDT could be considered instead. 

Interestingly, there were two patients who did not feel that the instructions in the dPDT@home kit were easy to understand or the materials easy to use and were not confident that they had performed the treatment correctly. This highlights the need for careful patient selection and active management and support.

## 4. Discussion

The comparison of dPDT@home patients’ clearance rates and that of those undertaking hospital-based dPDT may be compared to a small degree only, due to the differing sample sizes. As detailed above, five out of nine (56%) dPDT@home patients were reported as having good (>75%) clearance. This compares well with that of our previous study [8], where the same clearance was seen in 55% of hospital-based dPDT patients. Additionally, one out of nine (11%) dPDT@home patients were reported as moderate (50–75%) clearance, compared to 45% of hospital-based dPDT patients of the same study. The three out of nine (33%) of dPDT@home patients who were reported as poor (<50%) clearance all exhibited more severe AK, which is not unexpected, considering that our target patients are in the categories of mild-to-moderate AKs. Severe AKs may not respond as well to dPDT [13], but they typically benefit from additional preparation to the area, such as curettage. In-hospital, this is conducted by the healthcare professionals, whereas dPDT@home patients conduct curettage themselves, which may not be as vigorous.

Overall, the experience undertaking dPDT@home was positive. Patients highly rated the convenience of the dPDT@home kit, showed a strong willingness to have the treatment again, and highly rated the kit overall. Treatment response was also good in those with mild-to-moderate disease. However, it was clear that more severe disease did not clear as well with dPDT@home. This is as expected with daylight PDT, but the response was still disappointing compared with our previous experience with hospital-initiated daylight PDT [8]. One reason may be that hospital-initiated daylight PDT involves curettage of lesions, which shows increased PpIX accumulation compared to unprepared skin [14]. dPDT@home had less robust skin preparation pre-treatment and therefore potentially less effective removal of crusting from lesions, less uptake of PpIX, and therefore less effective outcomes in more severe disease. One option to improve outcomes may be to increase the drug incubation in more severe disease [15].

In both hospital and home-based dPDT, the healthcare professional is essential. In at-home dPDT, it was critical to have clear instruction, close telephone support, and follow-up with patients so that they were clear what was required, and so that they could ask any questions they may have. Without close support, it is our experience in other projects that outcomes are poor.

Patient selection is also critical. Time is spent by the healthcare professionals in conversation with the patient to decide together whether at-home dPDT is the right treatment for them (the realistic medicine approach described previously). As AK is a chronic disease, requiring long-term disease management, many patients have had multiple courses of dPDT over several years and are therefore more comfortable with self-administration. An example of another treatment for the same condition is topical application of 5-Fluorouracil (5-FU) [16]. The advantage of home-based daylight PDT is that it only needs to be undertaken 1–4 times per year, versus the repeated daily use of 5-FU.

As a regional secondary care service, we typically treat patients with more severe disease who have failed multiple other treatments before they reach us, and mild field-change AK will usually be managed in the community by primary care. dPDT@home treatment maybe best suited earlier in the patient journey, when disease is milder (i.e., in early-stage dermatology referral or in primary care). However, it was also clear from our study that higher patient satisfaction was correlated with higher patient support, and therefore very careful consideration would be required if testing the treatment closer to the community. Several issues were identified regarding the processes of patient selection, education, and follow-up arrangements. Patients would benefit from a dedicated support program.

## 5. Conclusions

In these feasibility and pilot studies, we utilized the principles of realistic medicine to work with patients in the development of and preliminary testing with the dPDT@home kit. The kit was refined as the studies progressed with emerging and important learning points on the delivery of a home-based treatment such as dPDT. We have demonstrated the principle of the kit, which was that successful dPDT can be performed at home with the right tools, clear instruction, careful patient selection, and support from the healthcare provider. These principles should now be explored in larger patient populations, ideally in randomized controlled trials.

The results from this pilot deployment of the dPDT@home kit have identified the types of individuals for whom this treatment is most suitable. However, it also underscores the necessity for very careful patient selection and comprehensive education, training, and support for both patients and clinicians to use the kit effectively. In hospital-based daylight PDT, patients typically attend the hospital for each treatment session, whereas with the dPDT@home kit, patients only needed to attend every second treatment. This demonstrates the potential of the dPDT@home kit to reduce the number of hospital visits required for patients requiring chronic AK disease management. Nevertheless, patients still require careful supervision and support to ensure optimal outcomes, which can be provided remotely through telephone contact.

The questionnaires’ results highlight the importance of setting patient expectations and of adopting a holistic approach to managing this chronic condition. In terms of sustainability, nearly all components of the dPDT@home kit are recyclable. The reduced number of hospital visits lowers patient travel, and the treatment’s reliance on natural daylight encourages the use of outdoor green spaces.

However, whilst patient acceptance of treatment was good, patient satisfaction with outcomes was less so, especially, we suspect in those with moderate-to-severe disease. This highlights the need for very careful selection and instructions and oversight in order to ascertain whether this mode of dPDT delivery may have a place in the management options for this common chronic and often challenging disease. It is premature to cast this aside, and it requires further careful evaluation, including objective efficacy and safety outcomes and health economics evaluation to determine its viability.

## Data Availability

Data from this publication can be sought from the corresponding authors upon reasonable request.

## References

[1] Dougherty T.J., Grindey G.B., Fiel R., Weishaupt K.R., Boyle D.G. (1975). Photoradiation Therapy. II. Cure of Animal Tumors With Hematoporphyrin and Light23. JNCI J. Natl. Cancer Inst..

[2] Agostinis P., Berg K., Cengel K.A., Foster T.H., Girotti A.W., Gollnick S.O., Hahn S.M., Hamblin M.R., Juzeniene A., Kessel D. (2011). Photodynamic therapy of cancer: An update. CA A Cancer J. Clin..

[3] Hamblin M.R., Hasan T. (2004). Photodynamic therapy: A new antimicrobial approach to infectious disease?. Photochem. Photobiol. Sci..

[4] Wiegell S.R., Wulf H.C., Szeimies R.M., Basset-Seguin N., Bissonnette R., Gerritsen M.J.P., Gilaberte Y., Calzavara-Pinton P., Morton C.A., Sidoroff A. (2012). Daylight photodynamic therapy for actinic keratosis: An international consensus. J. Eur. Acad. Dermatol. Venereol..

[5] McLellan L.J., O’Mahoney P., Khazova M., Higlett M., Ibbotson S.H., Eadie E. (2019). Ultraviolet radiation exposure during daylight Photodynamic Therapy. Photodiagnosis Photodyn. Ther..

[6] Wenande E., Phothong W., Bay C., Karmisholt K.E., Haedersdal M., Togsverd-Bo K. (2019). Efficacy and safety of daylight photodynamic therapy after tailored pretreatment with ablative fractional laser or microdermabrasion: A randomized, side-by-side, single-blind trial in patients with actinic keratosis and large-area field cancerization. Br. J. Dermatol..

[7] Dirschka T., Ekanayake-Bohlig S., Dominicus R., Aschoff R., Herrera-Ceballos E., Botella-Estrada R., Hunfeld A., Kremser M., Schmitz B., Lübbert H. (2019). A randomized, intraindividual, non-inferiority, Phase III study comparing daylight photodynamic therapy with BF-200 ALA gel and MAL cream for the treatment of actinic keratosis. J. Eur. Acad. Dermatol. Venereol..

[8] Cordey H., Valentine R., Lesar A., Moseley H., Eadie E., Ibbotson S. (2017). Daylight photodynamic therapy in Scotland. Scott. Med. J..

[9] Kotb I., Lesar A., O’Mahoney P., Eadie E., Ibbotson S.H. (2021). Daylight photodynamic therapy for actinic keratosis: Is it affected by the British weather?. Photodermatol. Photoimmunol. Photomed..

[10] Inns T., Mountain R. (2020). Designing ‘Realistic’ Healthcare Improvement. Des. Manag. Rev..

[11] McLellan L.J., O’Mahoney P., Logan S., Yule S., Goodman C., Lesar A., Fullerton L., Ibbotson S., Eadie E. (2019). Daylight photodynamic therapy: Patient willingness to undertake home treatment. Br. J. Dermatol..

[12] O’Mahoney P., Khazova M., Higlett M., Lister T., Ibbotson S., Eadie E. (2017). Use of illuminance as a guide to effective light delivery during daylight photodynamic therapy in the U.K.. Br. J. Dermatol..

[13] Wiegell S.R., Fabricius S., Gniadecka M., Stender I.M., Berne B., Kroon S., Andersen B.L., Mørk C., Sandberg C., Ibler K.S. (2012). Daylight-mediated photodynamic therapy of moderate to thick actinic keratoses of the face and scalp: A randomized multicentre study. Br. J. Dermatol..

[14] Bay C., Lerche C.M., Ferrick B., Philipsen P.A., Togsverd-Bo K., Haedersdal M. (2017). Comparison of Physical Pretreatment Regimens to Enhance Protoporphyrin IX Uptake in Photodynamic Therapy: A Randomized Clinical Trial. JAMA Dermatol..

[15] Heerfordt I.M., Wulf H.C. (2019). Daylight photodynamic therapy of actinic keratosis without curettage is as effective as with curettage: A randomized clinical trial. J. Eur. Acad. Dermatol. Venereol..

[16] Yentzer B., Hick J., Williams L., Inabinet R., Wilson R., Camacho F.T., Russell G.B., Feldman S.R. (2009). Adherence to a Topical Regimen of 5-Fluorouracil, 0.5%, Cream for the Treatment of Actinic Keratoses. Arch. Dermatol..

